# Targeted Nutrition in Chronic Disease

**DOI:** 10.3390/nu12061682

**Published:** 2020-06-05

**Authors:** Peter Bergman, Susanna Brighenti

**Affiliations:** 1 Department of Laboratory Medicine (Labmed), Division of Clinical Microbiology, Karolinska Institutet, ANA Futura, 141 52 Huddinge, Sweden; 2 Department of Medicine Huddinge (MedH), Center for Infectious Medicine (CIM), Karolinska Institutet, ANA Futura, 141 52 Huddinge, Sweden

**Keywords:** nutrition, chronic disease, prevention, inflammation, infection

Today, chronic disease is a major public health problem around the world that is rapidly increasing with a growing and aging population. This represents a diverse group of diseases that are broadly defined as conditions that last a year or more and require ongoing medical attention and/or restrict everyday life or certain activities [1]. Chronic disease includes, but is not limited to, various conditions such as cardiovascular disease, chronic lung disease, cancer, obesity, diabetes, kidney or liver disease, but could also involve chronic infections such as HIV and chronic inflammation as manifested in inflammatory bowel disease (i.e., Crohn’s disease and ulcerative colitis). It is well-established that our eating habits play an essential role in our general health and wellbeing and therefore poor nutrition is one of the most important risk factors for chronic disease. Consequently, while poor diet is a major contributor to chronic disease, there are significant clinical implications of nutritional supplementation and dietary recommendations for the targeted treatment of some of these diseases.

The present Special Issue on “Targeted Nutrition in Chronic Disease” includes three reviews [2,3,4] and seven original research articles [5,6,7,8,9,10,11]. A meta-analysis on coffee intake and obesity suggested that higher coffee intake was associated with modestly lower body mass index (BMI) and waist circumference in men but not in women [2]. The results from a previous meta-analysis support the favorable effects of coffee consumption in several chronic diseases and the consumption of three to four cups of coffee a day was associated with the largest risk reduction of various health outcomes [12]. There are several active compounds in coffee that could cause these health benefits, including anti-obesity effects, such as chlorogenic acid, caffeine, trigonelline and magnesium. 

Two review articles addressed the impact of specific diets on renal health and the development of chronic kidney disease [3,4]. In a narrative review, the findings from the 26 included studies suggested that in contrast to a “high fat, high sugar” diet, two of the recommended healthy diets, the DASH (Dietary Approaches to Stop Hypertension) and the Mediterranean diets, were associated with a lower risk of a poor renal outcome [3]. In another review, the authors summarized the evidence behind dietary therapy with nitrogen-free ketoacid analogues (KA) to prevent nutritional loss in the very low protein diets (VLPD) given to patients with chronic kidney disease [4]. Based on 35 randomized controlled trials and experimental studies, the results supported the beneficial effects of VLPD + KAs on renal function and the improvement of different metabolic parameters including acidosis, phosphorus, sodium intake, insulin resistance and bone metabolism [4]. However, the effectiveness of KAs, especially on uremic toxins and cardiovascular events, the dose and composition of the KA supplement, the cost-effectiveness and needs for dialysis, should be further investigated. 

In this Special Issue, two original articles involved a clinical trial [5] and follow up study [6] using nutritional supplementation with vitamin D (25(OH)D_3_) and the short chain fatty acid (SCFA) phenylbutyrate (PBA) to treat patients with slow progressive HIV infections. These are nutritional compounds with immunomodulatory properties and the ability to induce epigenetic changes in immune cells [13]. Prolonged inflammation is problematic in several chronic conditions, such as HIV infection, inflammatory bowel disease, diabetes, obesity and cardiovascular diseases. SCFA have anti-inflammatory effects and can reduce pathological inflammation while enhancing mucosal immunity [14]. HIV-1 infection is characterized by chronic immune activation and microbial translocation, which fuels viral replication and disease progression [15]. Dietary supplements such as vitamin D and PBA could restore nutritional status and enhance mucosal immunity. While vitamin D and PBA had no significant effects on HIV viral load or peripheral T cell counts or on BMI or middle-upper arm circumference (MUAC), vitamin D supplementation rapidly corrected a pre-existing vitamin D deficiency or insufficiency [5]. Vitamin D and PBA did not modulate gut-derived inflammatory markers or microbial composition in treatment-naïve HIV-1 individuals [6]. Low vitamin D levels are associated independently with a susceptibility to various clinical conditions including numerous infections [13]. Daily treatment with vitamin D and PBA was recently demonstrated to reduce clinical symptoms and adverse events in patients with pulmonary tuberculosis (TB), and nutritional supplementation was particularly effective in patients with moderate–severe TB disease and a vitamin D deficiency [16]. Interestingly, significantly lower vitamin D levels were found in COVID-19 patients who were polymerase chain reaction (PCR)-positive for SARS-Coronavirus-2 (SARS-CoV-2) compared with PCR-negative patients, indicating that vitamin D supplementation might improve vitamin D status and thus reduce the risk of SARS-CoV-2 infection [17]. 

Likewise, it has been suggested that vitamin K is involved in bone mineral density and chronic disease development. The bacterial flora in the gut may contribute to the maintenance of vitamin K, and in this Special Issue, Wagatsuma et al. demonstrated that vitamin K deficiency in patients with clinically inactive Crohn’s disease reduced the diversity of the gut microbiota [7]. The authors concluded that the composition of the gut microbiota was similar to that in patients with active Crohn’s disease [7]. These results warrant further study to investigate if nutritional supplementation could correct vitamin K deficiency and dysbiosis.

The alternative healthy eating index (AHEI) is a score that can be used to evaluate the risk and mortality of chronic diseases including kidney failure [18]. To improve the assessment of diet quality among hemodialysis (HD) patients, Van Duong and colleagues adapted and validated the AHEI by adding a group of eating variables to enhance the sensitivity of the score [8]. The inclusion of additional dietary information enhanced the performance of the AHEI–HD index [8], which could be used to provide dietary recommendations to hemodialysis patients and to obtain a better prediction of the mortality outcome in patients with a low AHEI–HD compared to patients with a high AHEI–HD. In another study on chronic kidney disease, the weekly consumption of 14 food intake markers was mapped in 839 Brazilian individuals with a self-declared diagnosis who were undergoing different treatment modalities [9]. The food patterns among patients with chronic kidney disease are diverse [19] and influenced by both sociodemographic and geographical variables [20]. The authors identified two dietary patterns, an “unhealthy” pattern (red meat, sweet sugar beverages, alcoholic beverages and sweets and a negative loading of chicken, excessive salt and fish) and a “healthy” pattern (raw and cooked vegetables, fruits, fresh fruit juice and milk) [9]. In comparison to the untreated reference group, the non dialysis-dependent and dialysis groups presented a low adherence to the unhealthy pattern whereas individuals who had undergone kidney transplants adhered to a better quality diet. 

An experimental study with relevance for chronic liver disease demonstrated that a major component in green tea, epigallocatechin-3-gallate (EGCG), can have protective effects against analgesic drugs (e.g., acetaminophen) that damage the liver via oxidative stress [10]. The protective mechanisms of EGCG were tested in a rat model and suggested to involve a reduced cytochrome P450 (CYP)-mediated bioactivation of acetaminophen, decreased hepatic oxidative stress and reduced acetaminophen-induced apoptosis and instead increased autophagy and reduced the accumulation of toxic products in the liver [10]. 

Finally, a small longitudinal pilot study investigated whether chemotherapy (oxaliplatin-fluoropyrimidine combination, XELOX) may cause protein loss and increase oxidative stress in 14 patients with advanced colorectal cancer [11]. There are several potential advantages for patients that result from the maintenance of a balanced amino acid composition during and after therapy. The results showed that XELOX therapy does not affect plasma amino acid levels or worsen oxidative stress [11]. However, these results need to be confirmed in larger studies, preferably with an interventional design in which specific amino acid mixtures targeting muscle protein synthesis and aerobic energy formation could be analyzed.

In conclusion, diet and nutrition are essential and game-changing components of our everyday lives and are therefore fundamental cornerstones of the prevention of chronic disease. The findings from the articles in this Special Issue may contribute to future recommendations about dietary patterns and supplementations, and they may provide ample opportunities for targeted nutritional interventions in chronic diseases that can be further tested in well-designed clinical trials.
